# Medical Jurisprudence of Insanity

**Published:** 1856-04-01

**Authors:** Forbes Winslow


THE JOURNAL

OF

PSYCHOLOGICAL MEDICINE

AND

MENTAL PATHOLOGY.

APRIL 1, 1856.

### Part First.

### Original Communications.

### Art. I.—MEDICAL JURISPRUDENCE OF INSANITY.

BY FORBES WINSLOW, M.D., D.C.L.

### Part I.—ON LUCID INTERVALS.

In the vast field of research connected with Judicial Psychology, there is no question of more importance in a metaphysical as well as in a medico-legal point of view than that which forms the subject of the present inquiry. Human life is often contingent upon a satisfactory solution of this subtle point, and the transmission of property to a great amount occasionally depends upon the answer given by the medical jurist to the question—Was there not associated with certain admitted conditions of mental disorder, lucid intervals,—a clear and distinct freedom of the mind from all delusions; such a repose and clearing up of the intellect as to enable the person to discriminate accurately between right and wrong, thus constituting him morally and legally responsible for his conduct, or rendering him competent to the exercise of a sound and rational judgment in the disposition of his property? In criminal as well as civil cases, the medical witness is often called upon to aid in the administration of justice by elucidating these abstruse points, and it therefore behoves him to be well acquainted, before entering the witness-box, with certain elementary or first principles, legal as well as psychological, in connexion with this subject, in order to be prepared to reply satisfactorily to interrogatories that may be addressed by counsel in the course of any judicial proceedings in which he may be professionally engaged, involving in their issue the existence or non-existence of lucid intervals.

It will be necessary for me, in the course of this exposition, to quote at length from some of the principal legal text-books and established medical authorities, and to cite the particulars of a few of the prominent and important cases in which this question has arisen in our courts of law. I propose, with a view to a full analysis of the literature of the subject, to consider—

I. Lunar Influence.
II. On the alleged Effect of the Moon on the Body and Mind.
III. On the Influence of Light on Vital Phenomena.
IV. The Origin of the Term Lunatic and Lucid Interval.
V. The Medical Acceptation of these Terms.
VI. The Legal Signification of these Phrases.
VII. An Examination of some of the Principal Civil and Criminal Cases involving in their Issue the Existence of Lucid Intervals.

### I.—Lunar Influence.

As the term *luna*-tic and lucid intervals are commonly supposed to be based on the hypothesis that the moon exercises a decided influence on the insane as well as upon various morbid phases of the intellect not amounting to derangement, it will be well primarily to discuss the much-vexed but interesting question, what is the effect of *lunar light*, not only upon the mind in health and disease, but upon the vital manifestations, vegetable as well as animal?

Let me, in the first place, refer briefly to the ancient opinions respecting the influence of the moon. From the earliest periods of antiquity, the idea generally prevailed, not only that the moon exercised a specific effect in the production and modification of disease, mental and bodily, but played a prominent and important part in the development of the character of nations, and in determining the destinies of the human race. Amongst the ancients, the moon was viewed as an object of superstitious regard. They held her in great religious and superstitious veneration, considering her influence superior even to that of the sun; in fact, they worshipped her as a deity. The new moons, or the first days of the month, were kept with great pomp and ceremony as national festivals. The people were obliged to rest on those days. The feast of new moons was a miniature of the feasts of the prophets. Eclipses, whether of the sun or moon, were looked upon as evidences of Divine displeasure. The Greeks consulted the different phases of the moon before contracting marriage; and the full moon, or the times of conjunction of sun and moon, was considered the most favourable periods for celebrating the ceremony, in consequence of the impression that the reproductive functions were under lunar influence.

"This connexion of the moon," says Dr. Laycock, "with the measure of time seems to have brought that planet into relation with the religious rites of ancient nations, as the Egyptians and Jews; and, also, to have given origin (in part) to the *mythological* idea so extensively prevalent of a lunar influence on marriage and child-bearing. Even the barbarous Greenlanders, as Egede informs us, believe in this superstitious notion. They imagine that the moon visits their wives now and then; that staring long at the full moon will make a maid pregnant, &c. Amongst the ancient nations the general idea was, that the lunar influence varied according to the age of the moon. Bubastis, the Egyptian Diana, was not equally favourable to parturient females and their offspring in her different phases. Amongst the Jews the full moon was believed to be lucky, and the two other disastrous."

"The full moon," says the Rabbi Abravanel, "is propitious to new-born children: but if the child be born in the increase or wane, the horns of that planet cause death; or, if it survive, it is generally guilty of some enormous crime.

"The Greeks and Romans entertained a similar idea respecting the lunar phases. The general opinion seems to have been, that the moon was propitious in proportion as its luminous face was on the increase. The ancient Greeks considered the day of the full moon to be the best day for marriage. Euripides makes Agamemnon answer, when asked on what day he intends to be married,

"Οταν Σελήνης εὐτυχὴς ἔλθῃ κύκλος." *

Hesiod asserted that the fourth day of the moon was propitious, but the eighteenth was bad, especially to females. Aristotle maintained that the bodies of animals were cold in the decrease of the moon, and that the blood and humours are then put in motion, and to those revolutions he ascribes the various derangements peculiar to women.

Lucilius, the Roman satirist, says that oysters and echini fatten during lunar augmentation, which also, according to Gellius, enlarges the eyes of cats; but that onions throw out their buds in the decrease of the moon, and wither in her increase, which induced the people of Pelusium to avoid their use. Horace also notices the superiority of shell-fish during the moon's increase.

Pliny takes notice of the same fact. He also adds that the streaks on the livers of rats answer to the days of the moon's

* "Iphigenia," act v. 717.

age; and that ants never work at the time of the lunar changes. He also informs us that the fourth day of the moon determines the prevalent wind of the month, and confirms the opinion of Aristotle that earthquakes generally occur about the new moon. Pliny asserts that the moon corrupts all dead carcases exposed to its rays, and produces drowsiness and stupor in those who sleep under her beams. He further contends that the moon is nourished by rivers, as the sun is fed by the sea. Galen asserts that all animals who are born when the moon is falciform, or at the half quarter, are weak, feeble, and short-lived; whereas those who come into the world during the full moon are healthy, vigorous, and long-lived.

Lord Bacon adopted the notion of the ancients. He maintains that the moon develops heat, induces putrefaction, increases moisture, and excites the motion of the spirits.* Van Helmont affirms that a wound inflicted during the period of moonlight is most difficult to heal; and he further says, that if a frog be washed clean and tied to a stake under the rays of the moon in a cold winter's night, on the following morning the body will be found dissolved into a gelatinous substance bearing the shape of the reptile, and that coldness alone, *without the lunar action*, will never produce the same effect.

The Spartans also considered the moon to have great influence, and no motive could induce them to enter upon an expedition, or march against the enemy, until the full of the moon. The Greeks and Romans believed that the moon presided over childbirth. The patricians of Rome wore the figure of a crescent upon their shoes, to distinguish them from the inferior order of men. The crescent was called *lulula*. Herodotus records that when the Lacedæmonians visited Athens, after the battle of Marathon, they waited until the moon had passed its full before they continued their march. (Erato. lxx.) The ancient alchymists attempted to localize planetary influences, maintaining that the *heart*, which represented, according to their physiological notions, the vital principle, was under the special protection of the *sun*; that the *brain* was regulated and controlled by the *moon*; that *Jupiter* presided over the *lungs*, *Mars* over the *liver*, *Saturn* over the *spleen*; that *Venus* took the *kidneys* under her kind control, and that *Mercury* sat in judgment upon the *reproductive functions*.

It will appear by the previously recorded data, that from the earliest periods in the history of the world the idea of the phenomena of organic life being subject to planetary control, was popular amongst enlightened and philosophic men. The following

* It is recorded that this great philosopher always had a severe attack of syncope at the time of a lunar eclipse.

passage proves that the great Roman satirist was bitten by this tradition :—

> "Ut mala quem scabies aut morbus regius urget,
> Aut fanaticus error, et iracunda Diana
> Vesanum tetigisse timent fugiuntque poetam,
> Qui sapiunt."*

I should be giving but an imperfect sketch of the literature of this subject if I were not to refer to the fact, that the poets, as well as philosophers and medical writers of ancient and modern time, had not failed to countenance by the authority of their genius the popular belief in the influence of the moon. Most of the great dramatists and epic poets have embodied in their immortal creations this idea.† The works of Shakspeare, Spenser, Beaumont and Fletcher, Ben Jonson, Milton, Byron, and Shelley, are replete with passages of exquisite beauty in relation to this subject. Our own imperishable bard, whose god-like apprehension and profound knowledge of the mind of man—whose intuitive insight into the subtle workings of the human heart and passions—whose intimate acquaintance with nearly every branch of knowledge and department of science, art, and philosophy, placed him like a bright and brilliant constellation on a giddy eminence far apart from the rest of mankind, has pointedly alluded to the moral influence of the moon on the human heart and intellect. In the "Twelfth Night," Viola apostrophizes Olivia as a

> "Most radiant, exquisite, and unmatchable beauty,"

"I heard you were saucy at my gates," replies Olivia, "and allowed your approach rather to wonder at than to hear you. If you be not mad, be gone; if you have reason, be brief: *'tis not that*

* Hor., "Ars Poetica."

† The moon appears to have called forth the fire and sublimity of poetic genius in all ages and in all climes. Some of the most beautiful and touching sonnets that adorn the English language are addressed to the moon. I cannot forbear (although it may not be considered quite *àpropos*) to quote an illustration from the pen of Charlotte Smith, one of our most exquisite writers of sonnets, the immortal Milton alone excepted :

> "Queen of the silver bow !—by thy pale beam,
> Alone and pensive, I delight to stray,
> And watch thy shadow trembling in the stream,
> Or mark the floating clouds that cross thy way ;
> And while I gaze, thy mild and placid light
> Sheds a soft calm upon my troubled breast :
> And oft I think—fair planet of the night—
> That in thy orb the wretched may have rest ;
> The sufferers of the earth, perhaps, may go—
> Released by death—to thy benignant sphere ;
> And the sad children of despair and woe
> Forget in thee their cup of sorrow here.
> Oh, that I soon may reach thy world serene !
> Poor wearied pilgrim in this toiling scene !"

*time of moon with me, to make one in so skipping a dialogue.*"

Again, in the play of "Antony and Cleopatra," Enobarbus, after entering Cæsar's camp, thus appeals to the moon:—

> "Be witness to me, O thou blessed moon!
> When men revolted shall upon record
> Bear hateful memory, poor Enobarbus did
> Before thy face repent."

After which, he adds, previously to expressing his deep contrition for his revolt against Antony—

> "*O sovereign mistress of true melancholy,*
> The poisonous damp of night disponge upon me:
> That life, a very rebel to my will,
> May hang no longer on me."

In "Othello," after the death of Desdemona, when Emilia enters the chamber to announce the foul murder of Roderigo by the hand of Cassio, the Moor, crushed to the earth by an accumulation of horrible misfortunes, exclaims in the agony of his soul, and in the bitterness of wild despair,

> "'Tis the very *error of the moon*,
> She comes more near the earth than she was wont,
> *And makes men mad.*"

In "King Richard the Third," the Queen, after rushing, whilst in a state of profound distraction, into the presence of the Duchess of York to announce the death of the King, passionately exclaims,

> "Give me no help in lamentation;
> I am not barren to bring forth laments;
> All springs reduce their currents to mine eyes,
> *That I, being govern'd by the watery moon,*
> May send forth plenteous tears to drown the world!"

Milton frequently alludes, in "Paradise Lost," to the pernicious effect of the moon. He speaks of

> "Demoniac frenzy, moping melancholy,
> And *moonstruck madness.*"

In Ben Jonson's "Alchemist," Tribulation says—

> "But how long time,
> Sir, must the saints expect?"

To which Subtle responds—

> "Let me see,
> How's the moon now? Eight, nine, ten days hence
> She will be silver potate; then three days
> Before to citronize,—some fifteen days."—Act iii. scene 1.

The notion of planetary influence has not been confined to classical regions, to classical authorities, or to the fanciful creations of the poet. It has existed amongst barbarous, uncivilized, and unlearned nations, who were profoundly ignorant of the views propounded by the ancient astrologers, or by the medical writers, who had somewhat engrafted the study of medicine upon that of astrology and astronomy. In referring to the alliance which formerly obtained between the two sciences, it has been well observed by an able writer and close observer of Nature, that no judicious person can doubt that the application of astrology to medicine, though it was soon perverted and debased till it became a mere craft, originated in *actual observations of the connexion between certain bodily affections and certain times and seasons.* Many, if not most, of the mischievous systems in physics and divinity have arisen from dim perception or erroneous apprehensions of some important truth; and not a few have originated in the common error of drawing bold and hasty inferences from weak premises.*

That the theory of planetary influence should have been advocated in early times, and have found zealous supporters, not only amongst the illiterate, but amongst learned and scholastic men, need excite no surprise when we consider how easily susceptible of demonstration is the fact of the moon's powerful effect in producing that regular flux and reflux of the sea which we call tides. Astronomers having admitted that the moon was capable of producing this physical effect upon the waters of the ocean, it was not altogether unnatural that the notion should become not only a generally received, but a popular one, that the ebb and flow of the tides had a material influence over the bodily functions. The Spaniards imagine that all who die of chronic diseases breathe their last during the ebb. Southey says, that amongst the wonders of the isles and city of Cadiz, which the historian of that city, Suares de Salazar, enumerates, one is, according to P. Labat, that the sick never die there while the tide is rising or at its height, but always during the ebb. He restricts the notion to the isle of Leon, but implies that the effect was there believed to take place in diseases of any kind, acute as well as chronic. "Him fever," says the Negro in the West Indies, "shall go when the water come low; him always come not when the tide high." The popular notion amongst the Negroes appears to be that the ebb and flow of the tides are caused by a *fever of the sea*, which rages for six hours, and then intermits for as many more.

* Southey.

### II.—On Lunar Influence in the Production of Bodily Disease.

I should be travelling out of my record, and be introducing much extraneous matter into this inquiry, if I were to discuss, at any considerable length, the alleged influence of the moon in the production of disease in general, independently of the supposed specific effect of lunar light upon the insane. The subject is one of *medical* as well as meteorological and philosophical interest, and cannot altogether be passed over in an inquiry like that under consideration. There exists extant in the writings of many able, truthful, and conscientious men a vast, body of valuable and indisputable evidence in support of the theory of planetary influence. We subjoin the names of the principal authorities on the subject:—Ballonius, Ramazzini,[1] Joubertus,[2] Joannes Morellus,[3] Mead,[4] Gemma,[5] Paræus,[6] Dr. Nicolas Fontana,[7] Dr. Cullen,[8] Dr. Balfour,[9] Dr. James Lind,[10] Dr. Jackson,[11] Dr. James M'Grigor,[12] Dr. James Gilchrist,[13] Dr. James Johnson,[14] Dr. Liddell,[15] Dr. Diemerbroeck;[16] and, in our own immediate epoch, Drs. Laycock[17] and Orton[18]—Dr. Laycock's Essays on the "Periodicity in the Phenomena of Life" and on the "Causes which determine Vital Phenomena," are able and ingenious, and will repay being read and studied by those disposed to investigate more fully this interesting topic—Dr. Milligan,[19] William Ramsay,[20] Dr. Prichard,[21] Arago,[22] and Dr. Lardner.[23]

Many of the great medical authorities of antiquity were clearly of opinion that the celestial bodies exercised a marked influence upon the bodily and mental functions. Dr. Haslam asserts that Hippocrates, whom he designates as a "philosopher and correct

[1] De Constitutionibus trium sequentium annorum, 1692, 1693, 1694, in mutinense civitate et illius Ditione, Dissertatio; which essay will be found in the first volume of his Opera Omnia Medica et Physiologica.

[2] On Epidemics.

[3] On Putrid Fever.

[4] De Imperio Solis et Lunæ in corpora Humana et Morbis inde oriundis.

[5] On the Plague of 1575.

[6] On the Plague.

[7] Observazione Sopra le Melattic che attacano li Europei nei climi Caldi, &c. Livorno, 1781.

[8] First Lines.

[9] Effects of Sol-Lunar Influence in Fevers. London, 1815.

[10] On Putrid Fevers.

[11] Treatise on the Connexion of the New and Full Moon with the Invasion and Relapse of Fevers.—*London Medical Journal* for 1787. Also, his Treatise on the Fever of Jamaica.

[12] Medical Sketches of an Expedition to Egypt.

[13] On the Diseases of India.

[14] On the Diseases of Tropical Climates.

[15] On the Diseases of Tropical Climates.

[16] On the Plague.

[17] Vols. ii. and iii., *Lancet*, 1842-3.

[18] On Cholera.

[19] Curiosities of Medical Experience.

[20] Astrologia Restaurata.

[21] Analysis of the Egyptian Mythology.

[22] Meteorological Essays.

[23] On Lunar Influence.

observer of natural phenomena," did not place any faith in the generally received notion respecting the influence of the moon. This is clearly an error. Hippocrates imbibed so strong a belief regarding the effects of the celestial bodies upon the vital manifestations, that he expressly recommends no physician to be entrusted with the treatment of disease who was ignorant of astronomical science; and he expressly advises his son, Thessalus, to study the science of numbers and geometry, affirming that the "rising and setting of the stars have great effect upon the distempers."*

The critical days, or *crises,* as they were termed, were said to correspond with the interval between the moon's principal phases.†

Galen adopted the Hippocratic notion. Hence the lunar periods were said by him to be connected with the exacerbation of particular diseases.

The doctrine of lunar influence has descended to modern times; and notwithstanding a section of the scientific world has altogether repudiated the idea, it has nevertheless found zealous advocates amongst the learned of all ages. Men of admitted judgment and sagacity have been found in the ranks of those who support this theory. At the threshold of this important and interesting inquiry it will be well to pause and consider, why any number of men of science should exhibit a disposition to discountenance this notion of planetary influence? I will let Dr. Orton, in the first place, answer this question.

"The difficulty of explaining lunar influence appears to be the great obstacle which in modern times has stood in the way of the belief of its existence and general prevalence. The ancients, who less minutely scrutinized the chain which connects effects with remote causes, implicitly believed in the existence of this power, simply because they saw the coincidence of its effects and certain states of the heavenly bodies, although they knew not that these bodies in other respects exert a physical influence on the earth. But since the progress of science has enabled men to trace more distinctly the manner in which changes arise from and produce other changes, this empirical mode of reasoning has ceased to be satisfactory; and the improvement of philosophy seems, in some instances, to have actually operated as a barrier to its further progress, by furnishing negative arguments against the existence of causes which we are unable to connect by any satisfactory theory with their effects. Every occurrence in Nature has been attempted to be accounted for on rational and general principles, and

* Epist. ad Thessalum de aëre, aquis, et locis.

† The crises which Hippocrates describes by the words *imperfecte judicabantur,* were, according to Dr. Balfour, nothing more than *intermediate inter-lunar crises;* and those to which he applies the terms *perfecte judicabantur,* were *final inter-lunar* crises.

it has been found much easier to deny than to explain the operation of the sol-lunar power. If, however, these principles were to be applied in all their extent to the other branches of medicine, they would strike at the very root of that imperfect science; for we know little more of the *modus operandi* by which ipecacuanha produces vomiting, or jalap produces purging, than we do of that by which the new or full moon produces attacks of intermittent fever, of mania, or epilepsy. We have the same kind of evidence of the agency of both these classes of causes; and after the proofs which have been adduced of sol-lunar influence, it would be nearly as preposterous to deny its existence—because we cannot account for it, because it does not produce its effects on all persons, or because the same occurrences frequently arise without its agency, as it would be to assert that a common dose of ipecacuanha or jalap will not produce vomiting or purging for precisely the same reasons. It does not, nevertheless, appear to be impossible to make some approach to the explanation of the nature of sol-lunar influence on known principles. It is proved, on the known laws of gravitation, that the various situations of the moon necessarily must have determinate effects on the atmosphere. Observations have shown that such is the case, and on these data considerable progress has already been made in the elucidation of this interesting subject.

"It appears to be very evident that sol-lunar influence is much more powerful within the tropics than in other parts of the world; and this may in some degree account for the little credit which it has met with; for little information, in comparison to the opportunities which are presented, has been conveyed from these countries to the native regions of philosophy. Dr. Balfour has indeed been impressed with all the importance of his subject, and even more than all; his situation and experience were such as to entitle his opinions to the highest attention, and he has given them to the world in the fullest manner; but he has failed in gaining a complete credit, probably from the dogmatical style which he has adopted, and from his having fallen into the error which is usually fatal to theorists—that of aiming at too much."*

Dr. Balfour's treatise will form the basis of some remarks when I come particularly to analyse the facts recorded by the different authorities relative to lunar influence in the production of disease. There has, I think, been a disposition to discourage of late years any minute, special, and scientific investigation of the facts recorded by men of veracity, on the presumption that the subject is altogether fanciful, visionary, and Utopian. If the question has been seriously considered with a view to elicit truth, has the inquiry been calmly and dispassionately pursued, and pursued, too, by competent observers, possessed of that preliminary amount of mathematical, astronomical, and meteorological science indispensably necessary in order to arrive at anything like a satisfactory result or scientific conclusion? I much doubt the fact.

* Dr. Reginald Orton. "Essay on the Epidemic Cholera of India," p. 202. 1831.

In general conversation on the subject, the observation is often made, "Oh, I have not overlooked the study of the subject; I have been careful to observe whether the moon does really exercise any influence in modifying the type of disease, and have arrived at the conclusion that the notion is a puerile and fallacious one." But when the question is asked as to the *mode of investigation* which has been adopted, it will generally be found to have been loose and unscientific. With undoubtedly a sincere disposition to arrive at the truth, the method adopted by the inquirer has not been sufficiently philosophic, logical, and exact as to entitle it to the respect of learned men. To establish the inconsistency displayed by writers on the subject, Dr. Orton cites passages from two standard works of scientific reference, relating to the subject of lunar light, in which the authors deny *in toto* its effects on the human organism. "The hypothesis of planetary influence," says one of the authorities, "has originated and passed by with the age of astrology."* Another writer remarks that, "as the most accurate and sensible barometer is not affected by the various positions of the moon, it is not thought likely that the human body should be affected by them." "But in the following page," says Dr. Orton, "*the writer furnishes a body of evidence to establish that the barometer has been found to be very remarkably affected by the various positions of the moon.*" It is not easy to reconcile such statements.

Before proceeding to analyse the facts cited by the authorities previously referred to, as illustrative of the influence of lunar light in the production of bodily disease, I would briefly direct attention to some of the well-known data regarding periodicity, as associated with the progress and type of disease. The theory of lunar influence is in a great measure based upon this well-established law. The doctrine of periodicity, as exhibited in the phenomena of life, is not of modern origin. The ancients were too close and accurate students and observers of Nature to have overlooked the fact. The phenomena of menstruation were the subject of particular observation in all ages, and its singular and well-marked periodical character was attributed to the operation of causes acting independently of those organic laws supposed to regulate the special functions of life. This periodicity is observed in a large class of febrile affections, particularly in the intermittent, remittent, and bilious fevers of tropical climates, in the class of disease termed *neuroses*, in all spasmodic and convulsive disorders, particularly in epilepsy and its allied affections, in many forms of insanity, and in the diseases classed under the term *exanthemata*. Dr. Laycock has entered so fully into the philosophy, physiology, and pathology of this subject that I

* Rees' "Encyclopædia." "Encyclopædia Britannica."

shall leave him in undisputed possession of the field. He has not, however, confined his remarks to the phenomena of periodicity as exhibited in disease, but has, with the hand of a master, traced the operation of the same law in the animal and vegetable creation, as well as in man in his normal and abnormal state. Dr. Radcliffe, in an unassuming but valuable work, has also touched upon this subject in an earnest, cautious, and philosophic spirit.* His chapter on Periodicity, natural as well as morbid, suggests to the physiologist and pathologist an important question —viz., whether its phenomena result from the operation of causes exterior to the body, or should be considered as the effect of certain laws of organic life yet undefined and unexplained by modern physiologists? Dr. Radcliffe agrees with the ancients, and with Dr. Mead and many of the moderns, in seeking for the causes of periodicity in sol-lunar influence, and he sees days, months, and years reflected in the lives of plants and animals; but he also considers this evidence in a new point of view, and elicits a new conclusion. In his opinion, this evidence shows that this sol-lunar influence is *necessary* to the life of animals as well as of plants, and most necessary, just in proportion as the vital principle loses that independency which is characteristic of the higher animal, and approximates to the dependency of the plant; and because it shows this, he concludes that all the changes which are found to take place in the sol-lunar influence *must* be accompanied by corresponding changes in vital manifestations. In other words, there must be signs of periodicity, and these signs must be most marked where the vital principle is least independent—in the plant more than in the animal, in woman more than man. For the same reason, he supposes there must be more marked signs of periodicity in cases where the vital energy is impaired by disease, and it is in this impairment, and in this only, that he thinks the true explanation of these signs is to be sought for. This is the lesson which Dr. Radcliffe deduces from this evidence. The question of lunar influence, indeed, is not specially gone into, but the whole tenour of the argument is to show that the moon *must* exercise a great influence upon the body.

"It would appear (says Dr. Radcliffe) that there are certain periodical changes in vital phenomena which reflect more or less distinctly the movements of the sun and moon, some of them corresponding to the day, others to the month, and others to the year; and that these changes are more and more conspicuous the lower the grade of organization in which they are displayed—more so in woman than in man—more in animals at the foot of the scale of being than in those at the summit—

* "On Epilepsy and other Affections of the Nervous System." By C. B. Radcliffe, M.D. 1854.

and most of all in the plant. Such is the conclusion which arises out of the physiological investigation of the question of periodicity. There can be no doubt as to the obscurity of the evidences of periodicity, even where that obscurity is least, as in epilepsy, and in affections allied to epilepsy; but there can also be no doubt as to the existence of these evidences. Thus on looking at a number of cases it is found that convulsions and spasm occur more frequently at night than in the day, more frequently about the time of new moon than about the time of full moon, and more frequently in the winter months than in the summer months. Of these evidences of diurnal, monthly, and annual periodicity, the diurnal are the most frequent and the best established; but all are sufficiently frequent and obvious to any one who will take the trouble to seek after them for himself."

There is much in the recorded facts and observations of Drs. Radcliffe and Laycock, as well as in the valuable treatises of Mead and Balfour, to strengthen the presumption that the periodicity referred to arises directly or indirectly from sol-lunar influences. Medical meteorology has not yet assumed the character and position of an exact and demonstrative science; and although I would concede much to those who have patiently considered this interesting branch of philosophic inquiry, I am in duty bound to pause before attributing too much power to those external agents (active I admit them to be) that are considered to regulate and control the great principle of life, either in its healthy or morbid manifestations. Can it be demonstrated that the vital law regulating the phenomena of menstruation acts independently of certain external stimuli? I repeat, is this fact susceptible of proof? Until we are satisfied that this important uterine function is not dependent upon a special law inherent in or acting specifically upon the uterus itself, shall we not be travelling beyond the limits of a safe and logical induction, by assuming as an indisputable and demonstrable fact, that the phenomena to which I refer are the effect of lunar conditions, or dependent upon certain meteorological states of the atmosphere induced by the physical aspects of the moon? I proceed with the historical analysis of the subject.

Dr. Mead's treatise, "De Imperio Solis et Lunæ in corpora Humana et Morbis inde oriundis," appeared soon after Sir Isaac Newton's immortal discoveries burst like a flood of dazzling light upon the world. Dr. Mead occupied a high position amongst the *literati* of Europe. His reputation as a scholar, as a physician, as a man of letters, and as a lover and cultivator of science, was universally established. He was the intimate friend of Pope, of Newton, and of Halley. He stood high in the estimation of foreign princes and kings, and the learned and scientific men of all countries eagerly sought his acquaintance, and felt honoured by his friendship. It is recorded in his biography that the King of Naples forwarded to Dr. Mead the two first volumes of Signor Bajardi's erudite work on the antiquities found in Herculaneum, paying him the compliment of asking in return a complete collection of his own works, and at the same time inviting him to his palace, for the purpose of showing him his valuable collection of Herculaneum antiquities. Considering the position of Dr. Mead, everything that fell from his pen was read with avidity, and his observations on all subjects were considered to be based upon a patient and accurate study of the great book of Nature. His work "De Imperio" was read with universal interest; and although it gave rise to much controversy, it nevertheless commanded the respect of his learned contemporaries. It was the first modern treatise on the subject, and proceeding from a physician of Mead's reputation, it at once formed the topic of general conversation and criticism. Such being the character of the work, I proceed briefly to analyse its contents.

I proceed to the analysis of Dr. Mead's essay. He, in the prefatory part of his treatise, dwells much upon the importance of a previous acquaintance with the mathematical principles of natural philosophy, in order fully to comprehend the subject of lunar influences.

He then attempts to demonstrate, in the first place, that the sun and moon, in proportion as they approach near the earth, independently of their influence upon heat and moisture, must, at certain times, materially modify vital phenomena. The author, in the second place, cites some facts illustrative of his theory, and then makes some suggestions in reference to the practical division of the subject.

Dr. Mead enters fully into the consideration of the effect of the moon on the winds, observing that the most boisterous seasons of the year occur about the vernal and autumnal equinox. It is a matter, he remarks, of common observation, that in the calmest weather there is some breeze at mid-day, at midnight, and also at full sea—that is, about the time the sun and moon arrives at the meridian, either over or under our hemisphere. Without entering more minutely into an analysis of Dr. Mead's able and ingenious essay, his theory of sol-lunar influence may be thus briefly epitomized:—According to Dr. Mead, the attraction of the sun and moon being increased at the *syzygies* (new and full moon), and the *perigees* (those situations in the moon's orbit in which she approaches nearest to the earth), and the passages over the equator, the weight of the atmosphere is consequently diminished, and it is rendered *mechanically unfit for respiration, and for supporting the due degree of pressure on the surface of the body.* Dr. Mead endeavours to establish, on

Newtonian principles, that in all the situations in which the sun and moon have been found to produce their greatest effects in raising the tides, rarefying and disturbing the atmosphere, and in producing disease, their joint attraction for the earth, or certain parts of it, is greatest ; and, on the contrary, where these effects are least evident, that these attractions are least. Dr. Mead maintains that the atmosphere is much more under lunar attraction than the ocean, owing to its greater height, which removes it further from the earth and nearer to the moon. Dr. Mead supposes that the influence of the moon is most visible in low conditions of vitality, and in certain states of disease, and its effects are more manifest on the nervous fluid than on the blood, or any other of the animal fluids. I consider it, however, fair that Dr. Mead should, to a certain extent, be the exponent of his own views ; and I therefore make no apology for quoting, *in extenso*, two important passages from his treatise, having special reference to his theory of lunar influence :—

"It has been now a considerable time since sufficiently made out that our atmosphere is a thin elastic fluid, one part of which gravitates upon another, and whose pressure is communicated every way in a sphere to any given part thereof. From hence it follows that if by any external cause the gravity of any one part should be diminished, the more heavy air would rush in from all sides around this part to restore the equilibrium which must of necessity be preserved in all fluids. Now this violent running in of the heavier air would certainly produce a wind, which is no more than a strong motion of the air in some determined direction. If therefore we can find any general cause that would at these stated seasons which we have mentioned, diminish the weight or pressure of the atmosphere, we shall have the genuine reason of these periodical winds, and the necessary consequences thereof. The flux and reflux of the sea was a phenomenon too visible, too regular, and too much conducing to the subsistence of mankind, and all other animals, to be neglected by those who applied themselves to the study of Nature. However, all their attempts to explain this admirable contrivance of infinite wisdom were unsuccessful till Sir Isaac Newton revealed to the world juster principles, and, by a truer philosophy than was formerly known, showed us how, by the united or divided forces of the sun and moon, which are increased and lessened by several circumstances, all the varieties of the tides are accounted for. And since all the changes we have enumerated in the atmosphere do fall out at the same times when those happen in the ocean, and likewise whereas both the waters of the sea, and the air of our earth, are fluids subject in a great measure to the same laws of motion, it is plain that the rule of our great philosopher takes place here—viz., that natural effects of the same kind are to be attributed as much as possible to the same causes.* What difference that known property of the air, which is not in water, makes in the case, I shall show anon.

* Newton, "Principia," p. 387.

Setting aside the consideration of that for the present, it is certain that, as the sea is, so must our air, twice every twenty-five hours, be raised upwards to a considerable height, by the attraction of the moon coming to the meridian; so that, instead of a spherical, it must form itself into a spheroidal figure, whose longest diameter, being produced, would pass through the moon. That the like raising must follow, as soon as the sun is in the meridian of any place either above or below the horizon; and that the moon's power of producing this effect exceeds that of the sun in the proportion of four and a half to one nearly. Moreover, that this elevation is greatest upon the new and full moons, because both sun and moon do then conspire in their attraction; least on the quarters, in that they then are drawing different ways, it is only the difference of their actions that produces this effect; lastly, that this intumescence will be of a middle degree at the time between the quarters and new and full moon, The different distances of the moon in her perigæum and apogæum likewise increase or diminish this power. Besides, the sun's lesser distance from the earth in winter is the reason that the greatest and least attraction of the air upwards more frequently happens a little before the vernal and the autumnal equinox. And in places where the moon declines from the equator, the attraction is greater and lesser alternately, on account of the diurnal rotation of the earth on its axis.

"Whatever has been said on this head is no more than applying what Sir Isaac Newton has demonstrated of the sea to our atmosphere; and it is needless to show how necessarily those appearances just now mentioned of winds, at the stated times, must happen hereupon. It will be of more use to consider the proportion of the forces of the two luminaries upon the air to that which they have upon the waters of our globe, that it may the more plainly appear what influence the alterations hereby made must have upon the animal body."

Dr. Mead then proceeds to demonstrate how much more powerfully the moon influences the atmosphere than the sea, and that the tides in the air, from lunar attraction, are much greater than on those of the ocean; and, after considering the effect of certain unnatural states of the atmosphere upon the barometer, and then the connexion between certain states of the barometer and special as well as epidemic diseases, he, in the subjoined passage, further develops his views as to the mechanical influence of certain conditions of the atmosphere on the respiratory organs:—

"It will not be difficult to show that these changes in our atmosphere at high water, new and full moon, the equinoxes, &c., must occasion some alterations in all animal bodies, and that from the following considerations:—

"First.—All living creatures require air of a determined gravity, to perform respiration easily and with advantage, for it is by its weight chiefly that this fluid insinuates itself into the lungs. Now, the gravity, as we have proved, being lessened by these seasons, a smaller quantity than usual will insinuate itself; and this must be of smaller force to comminute the blood and forward its passage into the left ventricle of the heart, whence a slower circulation ensues, and the secretion of the nervous fluid is diminished.

"Secondly.—This effect will be the more sure in that the elasticity of the atmosphere is likewise diminished. Air proper for respiration must be, not only heavy, but also elastic to a certain degree; for as this is by its weight forced into the cavity of the thorax in inspiration, so the muscles of the thorax and abdomen press it into the most minute ramifications of the bronchia in expiration; where, the bending force being somewhat taken off, and springy bodies, when unbended, exerting their power every way in proportion to their pressures, the parts of the air push against all the sides of the vesiculæ and promote the passage of the blood. Therefore the same things which cause any alterations in the property of the air will more or less disturb the animal motions. We have a convincing instance of all this in those who go to the top of high mountains; for the air is there so pure (as they call it)—that is, thin—and wants so much of its gravity and elasticity, that they cannot take in a sufficient quantity of it to inflate the lungs, and therefore breathe with great difficulty.

"Lastly.—All the fluids in animals have in them a mixture of elastic aura, which, when set at liberty, shews its energy, and causes those intestine motions we observe in the blood and spirits, the excess of which is checked by the external ambient air, while these juices are contained in their proper vessels. Now, when the pressure of the atmosphere upon the surface of our body is diminished, the inward air in the vessels must necessarily be enabled to exert its force in proportion to the lessening of the gravity and elasticity of the outward; hereupon the juices begin to ferment, change the union and cohesion of their parts, and stretch the vessels to such a degree as sometimes to burst the smallest of them. This is very plain in living creatures put into the receiver exhausted by the air-pump, which always first pant for breath, and then swell, as the air is more and more drawn out; their lungs at the same time contracting themselves, and falling so together as to be hardly discernible, especially in the lesser animals." *

Making due allowance for the obsolete terms used by Dr. Mead, as well as for the state of pathological and physiological science of his epoch, the reader will be able to detect, in the language which he adopts to enunciate the theory of lunar influence, the germs of some great truths, which have subsequently been confirmed, in all quarters of the globe, by appeals to the great book of Nature. Dr. Mead has undoubtedly laid himself open to the charge of attempting to prove too much; but are not all ardent and zealous cultivators of science exposed to the same imputation?

In the concluding part of the essay, Dr. Mead details a number of facts that have come under his own as well as the observation

* "Esperienze dell' Academia del Cimento," p. 118.

of his contemporaries, demonstrative of lunar influence. Some of the cases cited appear to have a somewhat fabulous origin; but making every allowance for some trifling and natural exaggerations into which the author has fallen, in his zealous endeavours to substantiate his pet theory, all who read his essay must admit that it is, to a great extent, based on a clear and accurate observation of facts, however loosely and inaccurately they may have, in a few instances, been recorded. It will be interesting, whilst glancing at the literary history of this subject, to refer to some of Dr. Mead's illustrations. Dr. Mead was physician to St. Thomas's Hospital during the time of Queen Anne's wars with France, and whilst occupying this honourable position, great numbers of wounded sailors were brought into the hospital. He observed that the moon's influence was visible on most of the cases then under his care. He then cites a case, communicated to him by Dr. Pitcairne, of a patient, thirty years of age, who was subject to epistaxis, whose affection returned every year in March and September—that is, of the new moon—near the vernal and autumnal equinoxes. Dr. Pitcairne's own case is referred to as a remarkable fact corroborative of lunar influence. In the month of February, 1687, whilst at a country seat near Edinburgh, he was seized, at nine in the morning, the very hour of the new moon, with a violent hæmorrhage from the nose, accompanied with severe syncope. On the following day, on his return to town, he found that the barometer was lower at that very hour than either he or his friend Dr. Gregory, who kept the journal of the weather, had ever observed it; and that another friend of his, Mr. Cockburn, professor of philosophy, had died suddenly, at the same hour, from hæmorrhage from the lungs; and also that six of his patients were seized, *at the same time, with various kinds of hæmorrhages*, all arising, it was supposed, from the effect of lunar influence on the condition of the barometer. Dr. Mead's essay is replete with cases illustrative of lunar influence analogous to those already cited. The practical part of the work I purposely leave untouched.

Having given the preceding sketch of Dr. Mead's essay, I now proceed to analyse Dr. Balfour's treatise, the second work of any importance specially devoted to this subject.

Dr. Francis Balfour's first dissertation was published in Calcutta, in 1784.* In 1790, in a "Treatise on Putrid Intestinal Remitting Fevers," published at Edinburgh, the periodical return of febrile paroxysms and their coincidence with the periodical intentions and remissions of sol-lunar power, which constitutes

* "Treatise on the Influence of the Moon in Fevers." This was subsequently reprinted in England, and also inserted in Dr. Duncan's "Medical Commentaries."

the foundation and proof of this theory, was investigated, described, and illustrated by two different plates, exhibiting a synoptical view of the whole system. The first part of that treatise is a regular logical synthesis, arising from facts observed and collected by himself to the discovery of certain prevailing tendencies in Nature, and thence to axioms or general laws. The second part is an analysis, in which these axioms or laws are employed to explain some of the most remarkable phenomena of fevers. The third part is an application of the principles of this theory to form general rules for practice.

This physician appears to have devoted great attention to the consideration of this subtle and disputed point in science; and, with a view to its satisfactory elucidation, placed himself in communication with all the medical men of note resident in our Indian presidencies, and elicited from them the result of their observations on the subject. Dr. Balfour maintains, that every type of fever prevalent in India is, in a remarkable manner, affected by the revolutions of the moon. Whatever may be the form of fever, he says that he has invariably observed that its first attack is on one of the three days which immediately precede or follow the full of the moon, or which precede or follow the change of the moon, so that the connexion which prevailed between the attack of the disease and the moon at or during the time referred to, was most remarkable; relapses in cases of fever are also said frequently to occur at such times. Dr. Balfour has observed, for a period of fourteen years, this tendency to relapse at the full and change; and, in particular cases, he was able to prognosticate the return of the fever at these periods with almost as much confidence as he could foretell the revolution itself. Putrid, nervous, and rheumatic fevers of India are, according to Balfour, equally under the influence of the moon. In attempting to explain these phenomena, Dr. Balfour says, that along with the full and change of the moon, there is constantly recurring some uncommon or adventitious state or quality in the air, which increases fever and disposes to an unfavourable termination or crisis; and that along with the intervals, there is constantly recurring a state or quality in the air opposite to the former, which does not excite but diminishes fever, and disposes to a favourable crisis.* Dr. Balfour has collected a vast body of valuable

* It will be well to state what Dr. Balfour means by a crisis. He defines a crisis to be "favourable changes which never fail to take place, in some degree or other, at the time of their *transition* from the lunar period in the inter-lunar interval, and generally on the first morning inter-meridional interval after it; at which juncture the maturity of the critical disposition concurs with the periodical decline of sol-lunar influence in bringing them about; and they are distinguished by one or more of the following symptoms—viz., a sediment, or particular turbid appearance, in the urine; a more free and natural perspiration; spontaneous

M 2

evidence in support of his lunar theory, establishing beyond all dispute that in tropical climates the regular diurnal and septenary changes observed in the character of the fevers of India, are coincident and correspondent with periodical sol-lunar conditions.

In the year 1783-4, Dr. Balfour had for many months the charge of a regiment of sepoys, of Cooch Behar, immediately under the vast range of mountains which separate the northern part of Bengal from Bootan. The prevalent diseases were fevers, or "fluxes" attended with fevers. During the first month four hundred men were invalided. The greater part, however, of these cases were convalescent in the course of the eight days that intervened between the full and change of the moon; but during the remaining months of his stay in that district, the diseases previously mentioned increased to almost double their extent at every full and change of the moon, falling down again to their former standard during the eight days which intervened between these two periods. With regard to small-pox occurring in India, Dr. Balfour expresses himself as perfectly satisfied that the full and change of the moon interfered with the eruption, and increased the accompanying fever to a dangerous degree.

The influence of the moon on the functions of life has been made the subject of observation and speculation in every part of India. The physiological and pathological effects of lunar light have been universally acknowledged by all medical men practising in tropical climates. The natives of India are taught to believe in lunar influence from early infancy. In the northern latitudes the effect of the moon's rays are said to be less sensibly felt than in India. In the latter country, those suffering from attacks of intermittent fever are often able to predict, by watching the phases of the moon, the accession of the disease. Balfour maintains, that the fact of diseases appearing during every day of the month is no legitimate argument against lunar influence.

"The human body," he says, "is subject to alterations from a thousand external physical circumstances as well as from many internal moral affections. These lay the foundation of disease at every period of life, but they do not overthrow the evidence of lunar influence, although they are apt to mislead with regard to effects that depend on that alone. The human body is affected in a remarkable manner by the changes of the moon. I am perfectly convinced, although I cannot constantly pretend to see the operation of the general law, nor to account at all times for its perturbation, and stools; and cleaner, moister, and softer tongue, with a more free and natural discharge of saliva, a more loose and copious expectoration, and a free discharge of bile, which seems to disappear and be suppressed in the course of the fever," &c.

agree in thinking that an attention to the power of the moon is highly necessary to the medical practitioner in India."

"It is a fact," says Dr. Orton, "which has been universally observed, particularly in tropical climates, that the moon has a great influence on the weather; the full and change tending to produce rain and storms, and the quarters being more frequently attended by fine weather." This is so well ascertained, and so thoroughly believed, at least in India, that it is nearly superfluous to adduce arguments or instances in support of it. On every side, then, we perceive the intimate connexion which exists between the three series of phenomena which have been noticed—the great lunar periods, disturbed states of the atmosphere, and the attacks of the epidemic. It will also be proved that the other principal circumstance which has been supposed to attend the prevalence of cholera, the depression of the barometer is likewise produced by the new and full moon. Dr. Orton says, "Sol-lunar influence is, doubtless, but one of the causes producing the state of the atmosphere which gives rise to cholera; and I have no doubt that the disease will often be found to make its appearance when the disturbing power of the sun and moon is least, and to subside when that power is at its height. General exacerbations of other epidemics, as well as of cholera, will usually be found to correspond to the moon's syzygies, and the remissions of the quarters."

Dr. Kennedy bears testimony, in his work on Epidemic Cholera, to the influence of the moon. He observes, "that the constitution here (India), both native and denizen, is assuredly under lunar influence, or, what is the same thing, under the influence of the changes of weather which invariably accompany the changes of the planet."

Diemerbroeck, in his well-known treatise on the Plague,* when speaking of the epidemic of 1636, says: "Two or three days before and after the new and full moon the disease was more violent; more persons were seized at these times than at others, and those who were then seized almost all died in a very few hours. Nescioquâ virium labefactione oppressi." In the dedication prefixed to this treatise, which is addressed to the prætor and consuls, and the whole senate at Utrecht, he thus describes the nature of his own situation, the opportunities he had of acquiring a knowledge of the disease, and his object in publishing the work :—

"As in all well-constituted states it is the duty of every one to contribute his advice and assistance for the public safety, that by

* "Isabrandi Diemerbroeck Montferto Trajectini, antehac Noviomagi, nunc Ultrajecti Medici de Peste." Libri Quatuor Dissertatio, &c. Arenaci, 1646.

their unanimous concurrence the present as well as impending evils of the state may be averted and repelled, I conceive that I should not act improperly if, concerning this plague, of all diseases the most cruel, and more destructful than an enemy, I, too, should offer some salutary advice toward the discovery of its hidden nature, together with some more certain method of curing it. For, as in warfare, none can so well elude the designs of the enemy, or repel his attacks, as one who has had experience in the art of war, so none can more effectually resist this cruel disease than one who has intrepidly opposed himself to its fury. This I did a long time ago, not only in the year 1633, when a most violent pestilential fever, the forerunner of this plague, afflicted most grievously the whole province of Gelderland, and principally the city of Nimeguen, where I was ordinary physician, and threw upon me so great a load of practice as hardly allowed me to take sustenance, but likewise, in 1636 and 1637, when the true plague raged so violently amongst the people of Nimeguen, and so great a number of sick was thrown upon my hands as to give me no rest or repose. Having at that time, with great danger, and at the risk of my life, investigated most inquisitively the nature of this most dreadful enemy, I now make public his *portrait*, delineated in this book, for the safety of all."

The same authority asserts, that during the epidemic fever which raged in Italy in 1693, patients died in great numbers on the 21st of January, at the period of the lunar eclipse. But as Dr. Lardner observes, when recording the fact, it may be objected that the patients who then died in such numbers at the moment of the eclipse might have had their imaginations highly excited, and their fears wrought upon, by the approach of that event, if popular opinion invested it with danger. That such an impression was likely to prevail is evident from the facts which have been recorded. In 1654, at the time of a solar eclipse, such was the strong opinion entertained on this subject, that patients in considerable numbers were ordered by their physician to be shut up in chambers well closed, warmed, and perfumed, with the view of escaping the injurious influence of the eclipse. The consternation that prevailed amongst all classes was very great, and such crowds rushed to the confessional, that the ecclesiastics found it impossible to exercise their spiritual vocations.

The authorities previously cited conclusively establish that lunar influence is not to be viewed as a mere myth, or as an Utopian speculation. A host of writers and observers confirm the fact beyond all disputation. It will remain for me to consider, not only the evidence in favour of the lunar theory, but the arguments advanced against the hypothesis. It is only by closely investigating both sides of the question that the philosopher in search of truth will be enabled to arrive at a safe deduction.

Before considering the main point under review—viz., the alleged effect of the moon upon the mind in a state of aberration—it will be necessary to revert to the supposed *modus operandi* of lunar light. In the first place, I will refer to the

### Morbid Phenomena of Lunar Light.

The morbid effect of the moon's rays upon the vegetable kingdom has long been the subject of observation and speculation. Many curious and apparently inexplicable facts are upon record illustrative of the phenomena. It will be well to refer to some of the more reliable data in connexion with this division of my subject. It is stated as a fact that if peas are sown in the increase of the moon they never cease blooming; that if fruits and herbs are set during the wane of the moon, they are not so rich in flavour, nor so strong and healthy, as when planted during the increase. M. Auguste de Saint-Hilaire states, that in Brazil, cultivators plant during the decline of the moon all vegetables whose roots are used as food; and, on the contrary, they plant during the increasing moon the sugar-cane, maize, rice, beans, &c., and in general those which bear the food upon their stocks and branches. Experiments, however, were made and reported by M. de Chanvalon, at Martinique, on vegetables planted at different times in the lunar month, and no appreciable difference in their qualities was discovered. There are some traces of a principle in the rule adopted by the South American agronomes, according to which they treat the two classes of plants distinguished by the production of fruit on their roots or on their branches differently; but there are none in the European aphorisms. The directions of Pliny are still more specific: he prescribes the time of the full moon for sowing beans, and that of the new moon for lentils. "Truly," says M. Arago, "we have need of a robust faith to admit, without proof, that the moon, at the distance of 240,000 miles, shall, in one position, act advantageously upon the vegetation of beans, and that, on the opposite position, and at the same distance, she shall be propitious to lentils." The wise husbandman is said to prune his vines in obedience to certain phases of the planet. It is a maxim amongst gardeners that cabbages and lettuces which are desired to shoot forth early, flowers which are to be double, trees which it is desired should produce early ripe fruit, should severally be sown, planted, and pruned during the decrease of the moon; and that, on the contrary, trees which are expected to grow with vigour should be sown, planted, grafted, and pruned during the increase of the moon. These opinions Dr. Lardner considers to be altogether erroneous. The increase or decrease of the moon, he maintains, has no appreciable influence on the phenomena of vegetation; and the experiments and observations of several French agriculturists, and especially of M. Duhamel du Monceau, have, he observes, clearly established this fact.

Mantanari has referred to physical causes for an explanation of the alleged lunar influence upon plants. During the day, he says, the solar heat augments the quantity of sap which circulates in plants, by increasing the magnitude of the tubes through which the sap moves, while the cold of the night produces the opposite effect by contracting these tubes. Now, at the moment of sunset, if the moon be increasing, it will be above the horizon, and the warmth of its light would prolong the circulation of the sap; but, during its decline, it will not rise for a considerable time after sunset, and the plants will be suddenly exposed to the unmitigated cold of the night, by which a sudden contraction of leaves and tubes will be produced, and the circulation of the sap as suddenly obstructed. This explanation does not satisfy Dr. Lardner, who remarks, that if it be admitted that the lunar rays possess any sensible calorific power, this reasoning might hold good, but it will have very little force when it is considered that the extreme change of temperature which can be produced by the lunar light does not amount to the thousandth part of a degree of the thermometer! Upon this point, however, philosophers are at variance. The lunar rays have, according to the experience of practical men, a decided calorific agency. The gardeners of Paris assured Arago that in the months of April and May they found the leaves and buds of their plants, when exposed to the full moon in a clear night, *actually frozen, when the thermometer in the atmosphere was many degrees above freezing-point.* He mentions these facts as proving that the moon's rays have a frigorific power, but that the largest speculums directed to the moon produced no such indications on a thermometer placed in their focus.* Dr. Howard, of Baltimore, has affirmed that, on placing a blackened upper ball of his differential thermometer in the focus of a thirteen-inch reflecting mirror, opposed to the light of the full moon, the liquor sunk, in half a minute, eight degrees!

Cases of sudden death and coma are recorded as resulting from improper and prolonged exposure to the intense light of the full moon. Sailors have been found dead on deck after sleeping under the moon's rays. It is also said that convulsions, apoplexy, epilepsy, and insanity have arisen from the same cause. Plutarch observes:—"Everybody knows that those who sleep abroad under the influence of the moon are not easily waked, but seem stupid and senseless."† Mr. Madden mentions that the Arabs attribute a morbific influence to the moon, and thinks

* Ferguss. "Bull. Univ." 1827, p. 383. † Plut. "Symp." B. 3.

it causes ophthalmia and catarrh. He thought there was some influence from it in the desert beyond the common dampness of the night.*

The questions that naturally occur to the mind in reference to the interesting inquiry under consideration are, whether the morbid phenomena alleged to result from the moon's rays are dependent upon the *mere intensity of lunar light*, or are to be considered as the effect of *some specific influence in the nature of the light itself?* Let me consider the first question. It is an admitted fact that the light of the full moon is at least 300,000 times more feeble than that of the sun. According to Cosmos, the mean distance of the earth from the sun is 120·32 times greater than the earth's diameter, therefore 20,682,000 German or 82,728,000 English geographical miles. The mean distance of the moon from the earth is 51,800 German or 207,200 English geographical miles.

It is said that the solar light reflected from the surface of the moon is in every zone fainter than the solar light reflected in the daytime from a white cloud.

Humboldt says, when speaking of this subject—" When taking lunar distances from the sun for determinations of geographical longitude, it is not unfrequently found difficult to distinguish the moon's disk amongst the more intensely illuminated cumuli. On mountains between 13,000 and 17,000 feet high, where in the clearer mountain air only light, feathery, cirrous clouds are to be seen, I found it much easier to distinguish the moon's disk; both being cirrous, from its slighter texture, reflects less of the sun's light, and the moon loses less in passing through the thin atmospheric strata." The ratio of the intensity of the sun's light to that of the full moon deserves further investigation, as Bouguer's generally received determination, $\frac{1}{300000}$, differs so strikingly from the indeed more improbable one of Wollaston, $\frac{1}{800000}$. Wollaston's comparison of the light of sun and moon, made in 1799, was based on the shadows cast by wax-light, while in the experiments with sirrus, in 1826-27, images reflected from a glass globe were employed. The earlier assigned ratios of the intensity of solar light as compared to that of the moon differs very much from the results here given. Michell and Enler, proceeding from theoretical grounds, have respectively concluded 450,000 and 374,000 to 1. Bouguer, from measurements of the shadows of wax-lights, had even made it only 300,000 to 1.

I think, after duly weighing the above facts, we must dismiss from the mind the impression that the intensity of the light of the moon, as compared to that of the sun, has anything to do with the supposed morbid effect of lunar light.

* "Travels in Turkey."

I proceed, in the next place, to the consideration of the questions, whether the alleged morbid effect of lunar rays is attributable to something specific in the composition of the light itself; and secondly, whether the supposed abnormal influence of the moon is not altogether owing to certain barometrical variations and meteorological phenomena consequent upon the phases or position of the planet? Is there anything specific in the composition of lunar light?

According to numerous observations which Arago made with his polariscope, the moon's rays contain *polarized* light. Polarized light *carbonizes*, and is therefore antagonistic to the sun's rays, which *oxygenate*. The light in the pent-up dwellings of large towns, inhabited by the poor, is polarized light, it being almost always only a reflected light; and the light and atmosphere contained in such dwellings, on the *mind* as well as the *body* of the inmates, is admitted to be destructive to vitality. The health of those exposed to such influences shows the want of oxygen; they suffer from venous torpidity, muscular debility, a circulation of carbonized blood, feebleness of the mind, a tendency to hallucination, delirium, sleeplessness, loss of muscular force, and inaptitude to work or engage in inactive employments.

What is polarized light? Sir David Brewster thus lucidly explains the phenomenon: When the ray of light falls on a transparent body, so as to be reflected from it, it is modified or affected in such a manner by this reflection, that upon meeting a second transparent body, it will either be reflected or not, according to the side which it presents to it. It will be reflected if it fall upon that body on either of the opposite sides, but will not be reflected if it fall upon either of the other two, at right angles to the former. Thus, suppose the ray, after being modified by the first transparent reflector, presents itself to the second, so as to be reflected, and call the side of the ray, on which it meets the second reflector, on the *north* side; if the second reflector is turned round, so that the *east* side of the ray meets it, there will be no reflection, and in like manner it will be reflected on the south and not on the west sides respectively. The same modification, whatever it may be, prevents the ray from being *doubly* refracted, by passing through Iceland crystal, which it meets on two of its opposite sides, but permits it to be doubly refracted by meeting the crystal on the two other sides. And this modification, with respect to double refraction, may be impressed upon the ray by a first double refraction, as well as by reflection from a transparent body. But where the modification is produced by reflection, it is most complete at one particular angle of incidence, which varies in different transparent substances.

Now, the existence of this phenomenon is certain; it is a fact that a change takes place in the ray by the operation of the first transparent body; it is a fact that this change has some kind of reference to the four sides of the ray, and affects those sides at right angles to each other differently. The observers of these appearances have explained them, by supposing that each particle of light has its adjacent sides endowed with opposite properties, and that the first reflecting, or double refracting body, turns or arranges all the particles of light in a ray, in such a manner that their similar sides are presented in the same direction to the second body. Now this arranging or turning of the particles, or this change operated by the first body upon the ray, whatever it may be, is termed, from analogy to the phenomena of magnetism —polarization.*

Having cursorily referred to two modes of explaining the phenomena of morbid lunar light, I have yet to consider the most rational and philosophic theory of lunar influence propounded—viz., the effect of the moon's position upon the *wind, temperature, and rain,* three meteorological conditions universally admitted to play an important part in the origin, spread, and modification of disease. It has been a vexed question with natural philosophers, whether the barometer is decidedly influenced by the phases of the moon. The facts illustrative of this point are too significant to justify a doubt upon the question.

A remarkable correspondence between the phases of the moon and certain states of the barometer has been observed by Luke Howard. This coincidence, he maintains, consists of a depression of the barometrical line on the approach of the new and full moon, and its elevation on that of the quarters. In above thirty out of fifty lunar weeks in 1790, the barometer was found to have changed its general direction once in each week, in such a manner as to be either rising or at its maximum for the week preceding, and following about the time of each quarter, and to be either falling, or at its minimum, for the two weeks about the new and full. It is remarkable that the point of greatest depression during the year—viz., 28·67, was about twelve hours after the new moon on the 8th of November, and that of the greatest and extraordinary elevation of 30·89, on the 7th of February, at the time of the last quarter. The variation from this coincidence seemed to be owing to an evident perturbation of the atmosphere. These observations were confirmed by observations made for ten years in the Royal Society's apartments. Mr. Howard supposes, therefore, that the joint attractions of the sun and moon at the new moon, and the attraction of the moon predominating over the sun's weaker attraction at the full, tend to depress the barometer by taking off the gravity of the atmosphere, as they

* "On Optics," by Sir David Brewster.

produce a high tide in the waters by taking off from their gravity; and again, that the attraction of the moon being diminished by that of the sun at her quarters, this diminution tends to make a high barometer, together with a low tide, by permitting each fluid to press with additional gravity on the earth. It is demonstrated *à priori* on the principles of the Newtonian philosophy, that the air ought to have its tides as well as the ocean, though in a degree as much less perceptible as is its gravity.* If this observation were strictly true, and the tides of the atmosphere were to those of the sea as the specific gravity of air is to that of water, the aërial tides must be extremely small, for the weight of air is very trifling compared to that of water. But it is known that the height of the tides of the sea bears some proportion to the extent of the sea, uninterrupted by land and to its depth. On both these accounts we should expect that the atmosphere would be more influenced by the moon's attraction than the sea, for it is vastly deeper and more extensive than the sea, and entirely unconfined.

Signor Tolado found that a greater elevation of the barometer takes place at the quarters than at the syzygies; it is less when the moon is in the northern signs than when in the southern. The mean diurnal height, which corresponds to the Tropic of Cancer, is less by a quarter of a line than that which corresponds to the Tropic of Capricon. It is one-sixth of a line less at the moon's perigee than at her apogee, and one-tenth of a line less at the syzygies than at the quarters; and there are vacillations in the mercury when the new or full moon corresponds to the apogean or perigean points. He found, also, that the perigee, the new and full moon, and the northern lunistice are favourable to bad weather; whilst the apogee, the quadratures, and the southern lunistice are more favourable to good weather.

Père Cotte, from observations of thirty-five years, found that the barometer had a tendency to descend at every new and full moon, and to ascend at the quarterly periods. He likewise found that the perigee and northern declination depressed the barometer, whilst the apogee and southern declination had the opposite effect.† Mr. L. Howard has satisfactorily established, that the moon's position, operating by the common effects of the attraction of gravitation, influences alike the course of the variable winds, the daily variations of the temperature, and the rain of any year; but not in every year alike, there being a constant periodical variation of the variation itself. The subjoined passage will illustrate Mr. L. Howard's views upon the point:—

* "Encyclopædia Britannica."

† Orton, on "Epidemic Cholera," p. 222; and "Lectures on Meteorology," by G. Luke Howard.

1. In 1807, the first year examined, the days on which a *northerly* wind appeared under a full moon (the spaces taken being weeks with the phase on the middle day) were double the number of those that occurred under the new moon; and the days on which a *south-west* wind blew under the new moon are to those under the full as thirty-three to seventeen. The *south-east* again, are six under new to four under full moon; while the *east* are eleven under full to five under new moon.

2. The rain of the year is found distributed accordingly—viz.

| | |
|---|---|
| For the weeks under last quarter . . | 6·92 in. |
| For those under new moon . . . . . | 5·09 „ |
| „ „ first quarter . . . . . | 6·17 „ |
| „ „ full moon . . . . . . | 0·84 „ |

The total for the solar year being 19·02 in., we find that *not a twentieth part of the rain of the year fell in that quarter of the whole space which occurred under the influence of the moon at full.*

3. Contrary to the state of the *barometrical variation* in 1798, almost all the principal elevations of the column appear in this year under the full moon, along with the northerly winds.

4. Lastly, the *mean temperature* of the weeks preceding new and full moon is lower in this year by two degrees than that of the weeks preceding the quarter.

(*To be continued.*)

---

